# Excessive computer use as an oral health risk behaviour in 18‐year‐old youths from Poland: A cross‐sectional study

**DOI:** 10.1002/cre2.183

**Published:** 2019-05-01

**Authors:** Dorota Olczak‐Kowalczyk, Jacek Tomczyk, Dariusz Gozdowski, Urszula Kaczmarek

**Affiliations:** ^1^ Department of Paediatric Dentistry Medical University of Warsaw Warsaw Poland; ^2^ Department of Human Ecology Cardinal Stefan Wyszynski University Warsaw Poland; ^3^ Department of Experimental Design and Bioinformatics Warsaw University of Life Sciences Warsaw Poland; ^4^ Department of Conservative and Paediatric Dentistry Medical University of Wroclaw Wroclaw Poland

**Keywords:** adolescents, computer use, oral health behaviour

## Abstract

**Background:**

Many studies have indicated that the excessive use of computers (more than 3 hr/d) might be associated with an unhealthy life‐style.

**Aim:**

The aim of this study was to evaluate the relationship between excessive computer use with the condition of the teeth and periodontium and the oral health behaviour of 18‐year‐olds.

**Design:**

Cross‐sectional studies, using a questionnaire, were carried out on 1,611 18‐year‐olds from Poland. The questionnaire contained questions about socioeconomic status and information about health‐related behaviour. The condition of their teeth and gingivae were clinically assessed.

**Results:**

Excessive (>3 h/d) computer use was reported by 492 (31%) of participants, who had an increased frequency of unfilled cavities (1.97 vs. 2.27, *p* = .047) and a higher risk of oral hygiene neglect (e.g., using dental floss 41% vs. 34%, *p =* .009). Excessive computer use was also seen to be associated with poor dietary habits. Individuals who declared excessive computer use also had a higher risk of gingival bleeding (35% vs. 29%, *p* = .009).

**Conclusion:**

In the group studied, excessive computer use by adolescents constituted a risk factor for neglect of oral hygiene, poor dietary choices, and failure to benefit from oral health care. Therefore, these aspects should be included in the risk assessment of oral disease and incorporated into educational programs that promote a healthy lifestyle.

**WHY THIS PAPER IS IMPORTANT TO PAEDIATRIC DENTISTS**

The results confirm the negative effects of excessive computer use on oral health behaviour and on the oral cavity in general.

These results highlight the important role of paediatric dentists in transferring knowledge to adolescents that excessive computer use increases the risk of changes in the oral cavity. This information should be taken into account when designing educational programs for adolescents promoting a healthy lifestyle.

## 1 INTRODUCTION

Adolescence is a complex and difficult stage in human development in which psychological stress associated with physical and social changes is accompanied by an increase in emotional reactivity. The desire to escape from the close supervision of parents and the need to “experiment” contributes to the use of pathological (dangerous) methods to release tension, boost mood, or facilitate communication with peers. Common risky behaviours of adolescents include cigarette smoking, drinking alcohol, taking drugs, and following inappropriate diets. (Hoffman, Austin, Pinkleton, & Austin, 2017; Marshall, Biddle, Gorely, Cameron, & Murdey, 2004; Panatto et al., 2013; Schinke, Fang, Kristin, & Cole, 2008) Such behaviour often persists into adult life, affecting the health, including the oral health, of a person.

Similarly, addiction to the excessive and uncontrollable use of computers (e.g., Internet and computer games), which can interfere with a person's daily life and may also have a negative impact on the oral health. Many studies indicate that the excessive use of computers (>3 h/d), including video games, might be associated with an unhealthy life‐style such as a sedentary life, irregular meals, junk food habits, and a lack of sleep.(Kim et al., 2010; Peltzer, Pengpid, & Apidechkul, 2014) Such behaviours appear to be associated with many health problems including physiological problems (e.g., childhood obesity and diabetes) and/or psychological problems (e.g., violence, aggressive behaviour, and self‐body image issues).(Mark, Boyce, & Janssen, 2006; Marshall et al., 2004) Problematic computer use has been reported by several authors.(Hawi, 2012; Siomos, Dafouli, Braimiotis, Mouzas, & Angelopoulos, 2008; Tsitsika et al., 2012) In 2006, among Canadian youth, only 41% of girls and 34% of boys spent 2 h/d or less in front of a screen (TV or computer).(Mark et al., 2006) Similar periods of daily computer use have been reported in the United States, where one study found that only 37% of girls and 34% of boys spent 2 h/d or less in front of the screen.(Roberts, Foehr, Rideour, & Brodie, 1999) This means that the majority spent more than 2 h/d in front of the screen.(Roberts et al., 1999) A study conducted in Poland (in the northeastern region) suggested that nearly 23% of respondents spent about 2 h/d on the computer, 20% spent 3 h/d, 15% spent 4 h/d, and 13% spent 5 h/d.(Lićwinko, Krajewska‐Kułak, & Łukaszuk, 2011) Generally, this suggests that the average youth spends about 50 hr in front of the computer per week.(Tomczyk & Kopecký, 2016) It is perhaps unsurprising that the American Academy of Pediatrics has released guidelines recommending that “screen time” in front of a television or computer in children and adolescents should be limited to no more than 2 h/d.(American Academy of Pediatrics (AAP), 2001) The World Health Organization recently classified computer gaming as an official mental disorder, characterized by impaired control over gaming and an increased priority given to gaming over other activities in life.(World Health Organization, 2018)

Although there appears to be an important relationship between the excessive use of computers and oral health, it is not a very common subject for studying. Such studies have been conducted in Korea, due to fact that this country has the highest proportion of the Internet users in the world.(Kyung‐Yi & Kang‐Soon, 2018; Park & Lee, 2018) Giving the high prevalence of the excessive use of computers in Polish adolescents and a lack of previous study in this area, the current study investigated the association of excessive use of computers with oral health status in a sample of Polish adolescents.

The aim of the study was to test the hypothesis, that spending too much time in front of the computer can affect not only health habits but also changes in the health of the teeth and periodontal tissues. This aim was realized by answering the following questions:

1. Whether excessive computer use affects oral hygiene?
2. Whether excessive computer use affects dietary habits?
3. Whether gender and/or socioeconomic status (SES) can affect the relationship between excessive computer use with oral health and dietary behaviours?

The study was limited to a population of 18‐year‐olds from all regions of Poland.

## 2 MATERIALS AND METHODS

The study was approved by the Bioethics Committee at the Medical University of Warsaw (Poland; KB/134/2017 of the 6th of June 2017). All participants signed written consent forms before taking part in the study.

**Figure 1:**
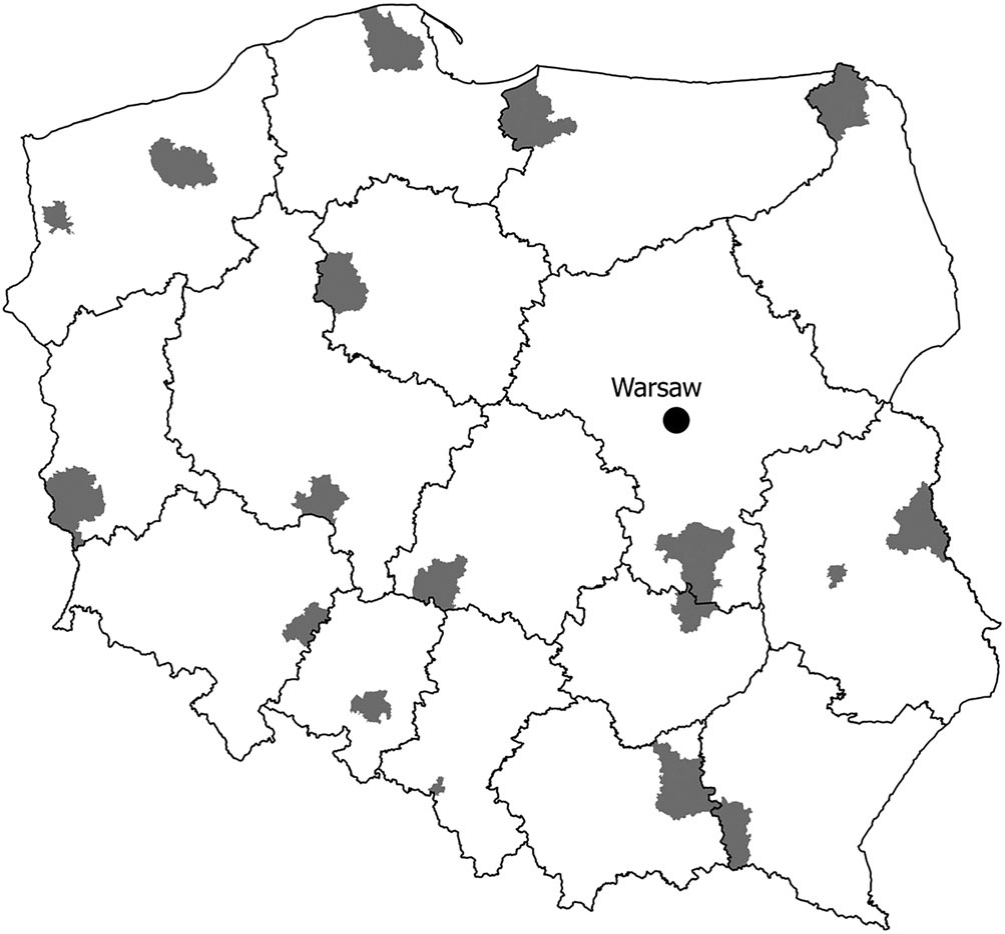
Map of Poland with marked areas covered by the research

The study took place in 2017. It was cross sectional and was within the programme of the Ministry of Health, titled “Monitoring the oral health of the Polish population.” It was limited to 18‐year‐olds, attending vocational and secondary public schools located in all 16 regions of Poland. From each region, either one province, which had an urban–rural character, or two provinces, one of which was typically urban and the other a typical rural one, were selected. Thus, the population studied came from, both rural and urban, areas of each Polish region (Figure 1). According to the economic data from 2014, the average monthly income per capita in Polish rural households was 1051 PLN (around 250 EUR) and, in cities, was 1516 PLN (around 352 EUR).(Łącka, 2017) As public schools dominate in Poland, pupils attending them represent different social classes. Schools were selected in a multi‐layered grouping (district, commune, and city). Pupils from schools whose directors agreed to participate in the study were invited to participate in the study. The study included pupils present on the day that the dental examiners visited heir schools and who had given their written permission to participate in the study. The size of the study group was estimated based on data from the Polish Central Statistical Office, which indicated a population size of 18‐year‐olds as 390,602. Unfortunately, because a dozen school heads declined to allow their schools to participate in the study, in some cases, supplementary research had to be carried out. The study began in November 2017 and was completed in 3 weeks to try to ensure consistency. The supplementary research was performed no later than three weeks after the first research.

A total of 1,611 18‐year‐olds (764 males and 847 females) were enrolled in the study, including individuals from both urban and rural regions of Poland. All individuals were asked to complete a questionnaire (Data S1) and a clinical oral examination (Data S2) following the WHO guidelines.(World Health Organization. Oral health surveys: basic methods. Geneva: World Health Organization, 2013) The questionnaire covered the following:

1. socioeconomic status (SES) (parents' level of education and financial status of the family),
2. the number of hours of daily computer use (more than 3 hr of daily computer use was considered excessive, not including during school hours),
3. oral hygiene habits (frequency of tooth brushing, using dental floss, and dental appointments for a routine dental check‐up at least once per year),
4. dietary habits (number and type of meals, frequency of consumption of food and drink such as fresh fruit and vegetables, sweet fizzy beverages, sugar‐free fizzy beverages, sweetened juices, and crisps), and
5. oral pain and absence from school due to oral pain.

The condition of the teeth and periodontium in each quadrant of the dentition was clinically examined under artificial lighting, using a WHO 621 probe.(World Health Organization. Oral health surveys: basic methods. Geneva: World Health Organization, 2013)

The number of teeth and decayed surfaces, the number of teeth extracted, and the number of filled teeth were recorded, the decayed, missing, and filled teeth (DMFT) and the decayed, missing, and filled surfaces (DMFS) indices were calculated. The presence or absence of gingival bleeding after probing and probing pocket depth around each tooth was assessed. Clinical examinations were performed by general dental practitioners following prior training and calibration.

### 2.1 Statistical methods

The Cohen's kappa coefficient between the reference examiner and the other examiners regarding the condition of the teeth and periodontium ranged from 0.857 to 1.000, which constituted a high inter‐examiner reliability.

The comparison of the means for quantitative variables between the two groups (males vs. females and computer usage ≤3 h/d vs. > 3 h/d) was carried out using a *t* test. Although comparison of the percentages for categorical variables between these groups was performed using Pearson's chi‐squared test, The assessment of the association between computer use (transformed into a binomial variable, i.e., computer usage ≤3 h/d vs. > 3 h/d) and other variables (including hygiene‐related behaviour, dietary habits, and oral health) was performed using simple and multiple logistic regression. Socioeconomic factors (SES) were considered as covariates in the regression analysis. To avoid a confounding effect of the other variables, the results were presented as an odds ratio (OR) for simple regression and an adjusted odds ratio (AOR) for multiple regression where socioeconomic factors were included in the statistical models as covariates. The confounding factors for calculating AOR were selected based on Spearman's correlation coefficient. Statistical analysis was performed using Statistica 13 software (http://www.tibco.com). In all analyses the statistical significance value was set at *p* < .05.

## 3 RESULTS

The directors of 47 of the 60 schools, who were invited, agreed that their schools could participate in the study. Due to a lack of consent or a wrongly completed questionnaire, out of over 3,000 pupils, 1,611 were finally included in the study. A power calculation had indicated that the minimum sample size required for this study was 1,531 assuming the margin of error was not higher than 2.5% for the binomial proportion (for any prevalence of the dental problems) at a 95% confidence level. This meant that the group of 18‐year‐olds was random and was representative of all Polish 18‐year‐olds. When the results were anlysed that the majority of the OR values obtained were close to the AOR. This means that socioeconomic factors had only slightly modified the impact of computer use. At the same time, it was an indirect proof that there was no explicit segmentation of the sample; that is, it was a varied random sample but without any strong divisions into the clearly different subgroups, which indirectly confirmed that the selected sample represented the population of Polish 18‐year‐olds. Approximately one third (31%, 492/1611) spent over 3 h/d on the computer with a significantly higher number of males (38%) than females (24%). On average, the males spent 3.5 h/d in front of the computer, whereas the females spent an hour less (2.7 h/d; Table 1).

**Table 1:** Significant differences in health‐related behaviour and food consumption in the study group according to gender

Habits	Male	Female	Total
>3 h/d spent on the computer	293/764 (38%)	199/847 (24%)	492/1611 (31%)
	p < 0.001^b^		
Average number of hours of computer usage^a^	3.47 ± 2.42	2.72 ± 1.82	3.8 ± 2.16
	*p* < .001^b^		
Tooth brushing at least twice a day	403/764 (53%)	693/847 (82%)	1096/1611 (68%)
	*p* < .001^b^		
Using dental floss	215/764 (28%)	415/847 (49%)	630/1611 (39%)
	*p* < .001^b^		
Consumption of more than 3 snacks during the days	145/764 (19%)	95/847 (11%)	240/1611 (15%)
	*p* < .001^b^		
Consumption of fresh fruits and vegetables every day or a few times a day	338/764 (44%)	459/847 (54%)	797/1611 (49%)
	*p* < .001^b^		
Consumption of sweet fizzy drinks every day or a few times a day	208/764 (27%)	148/847 (17%)	356/1611 (22%)
	*p* < .001^b^		
Consumption of crisps every day or a few times a day	91/847 (11%)	55/764 (7%)	146/1611 (9%)
	*p* < .001^b^		

a *x* ± *SD*.

b Statistical significance *p* < .05.

Females demonstrated good oral hygiene care (tooth brushing, using dental floss, and following a diet containing fruits and vegetables) more frequently than males. Males expressed a preference for unhealthy eating habits (consumption of sweetened juices, fizzy drinks, and crisps) significantly more than females. Correlations between the sexes and oral health behaviour/dietary habits were statistically significant (*p* < .001; Table 1).

A simple logistic regression analysis indicated that excessive computer use was associated with poor oral health behaviour and an increased consumption of unhealthy food (Table 2). The AOR model confirmed that excessive computer use increased the risk of brushing teeth less than twice a day (AOR = 0.71, *p* = .004) and/or a less frequent attendance for dental appointments (AOR = 1.42, *p* = .035), although the AOR model indicated that the factors significantly affecting the relationship between using dental floss and excessive computer use were those related to SES (AOR = 0.87, *p* = .240; Table 2).

**Table 2:** Relationship between excessive computer use, health‐related behaviour, and food consumption

Habits	>3 h/d	≤3 h/d	OR	p	CI^a^	AOR	p	CI^a^
Tooth brushing at least twice a day	292/492 (59%)	800/1119 (72%)	0.58	.001^b^	0.47	0.71	.004^b^	0.56
					0.73			0.90
Using dental floss	167/492 (34%)	457/1119 (41%)	0.74	.009^b^	0,60	0.87	.240	0.69
					0,93			1.10
No dental appointment within last 12 m	69/492 (14%)	115/1119 (10%)	1.42	.030^b^	1.04	1.42	.035^b^	1.03
					1.96			1.96
Dental appointment due to toothache	96/492 (20%)	209/1119 (19%)	1.06	.694	0.81	1.07	.610	0.82
					1.38			1.41
Follow‐up dental appointment	195/492 (40%)	454/1119 (41%)	0.96	.724	0.77	1.00	.977	0.80
					1.19			1.24
Missed breakfast	98/492 (20%)	160/1119 (14%)	1.49	.005^b^	1.13	1.54	.003^b^	1.16
					1.97			2.04
Fresh fruit/vegetables	221/492 (45%)	578/1119 (52%)	0.76	.013^b^	0.62	0.82	.072	0.66
					0.94			1.02
Sweet fizzy beverages	127/492 (26%)	219/1119 (20%)	1.43	.005^b^	1.11	1.30	.044^b^	1.01
					1.84			1.68
Sugar‐free fizzy beverages	92/492 (19%)	158/1119 (14%)	1.40	.020^b^	1.06	1.36	.035^b^	1,02
					1.86			1.81
Sweetened juices	115/492 (23%)	207/1119 (19%)	1.34	.025^b^	1.04	1.30	.047^b^	1.00
					1.74			1.69
Crisps	60/492 (12%)	81/1119 (7%)	1.78	.001^b^	1.25	1.63	.008^b^	1.14
					2.53			2.33

Abbreviations: AOR, adjusted odds ratio for socioeconomic factors; CI, confidence interval; OR, odds ratio.

a CI: lower and upper bound, 95%.

b Statistical significance *p* < .05.

A similar analysis was carried out considering food consumption. Young people who use their computers excessively frequently missed breakfasts, consumed less fresh fruit and vegetables, and admitted to more frequent consumption of crisps, sweetened juices, or fizzy beverages. Even after adjusting for socioeconomic status (AOR), the risk of unhealthy eating habits was higher in adolescents who used the computer excessively (Table 2).

Adolescents of both sexes who did not use the computer excessively (≤3 h/d) frequently brushed their teeth more (at least twice a day) than those who spent more than 3 h/d in front of the screen (males *p* < .001, females *p* = .035). However, using dental floss or attending follow‐up dental appointments did not show any significance between the sexes (Table 3). A similar tendency was observed during the analysis of dietary habits. Adolescents of both sexes who spent more than 3 h/d in front of the computer more frequently did not eat breakfast (males *p* = .029, females *p* = .036), although other eating habits have proved important only among females (Table 3). Females spending more time (>3 h/d) in front of the computer more often showed unhealthy eating habits than females who did not use the computer very frequently per day (consumption of sweet fizzy beverages 26% vs. 14%, sweetened juices 26% vs. 16%, and crisps 13% vs. 5%; Table 3).

**Table 3:** Relationship between excessive computer use, health‐related behaviour, and food consumption according to gender

Habits	Sex	>3 h/d	≤3 h/d	χ ^2^	p
Tooth brushing at least twice a day	Male	140/293 (48%)	262/471 (56%)	15.61	.001^a^
	Female	152/199 (76%)	538/648 (83%)	4.45	.035^a^
	*χ* ^2^	40.18	100.46		
	*p*	.001^a^	.001^a^		
Using dental floss	Male	73/293 (25%)	136/471 (29%)	1.43	.233
	Female	94/199 (47%)	321/648 (50%)	0.32	.570
	*χ* ^2^	26.33	48.20		
	*p*	.001^a^	.001^a^		
No dental appointment within last 12 m	Male	38/293 (13%)	51/471 (11%)	0.80	.370
	Female	31/199 (16%)	64/648 (10%)	4.97	.026^a^
	*χ* ^2^	0.67	0.27		
	*p*	.414	.605		
Dental appointment due to toothache	Male	53/293 (18%)	84/471 (18%)	0.01	.929
	Female	43/199 (22%)	125/648 (19%)	0.51	.473
	*χ* ^2^	0.93	0.38		
	*p*	.334	.537		
Follow‐up dental appointment	Male	114/29 (39%)	180/471 (38%)	0.01	.929
	Female	81/199 (41%)	274/648 (42%)	0.16	.693
	*χ* ^2^	0.16	1.87		
	*p*	.689	.171		
Missed breakfast	Male	55/293 (19%)	61/471 (13%)	4.75	.029^a^
	Female	43/199 (22%)	99/648 (15%)	4.37	.036^a^
	*χ* ^2^	0.60	1.20		
	*p*	.439	.272		
Fresh fruit/vegetables	Male	126/293 (43%)	212/471 (45%)	0.30	.587
	Female	95/199 (48%)	366/648 (56%)	4.69	.030^a^
	*χ* ^2^	1.07	14.37		
	*p*	.300	.001^a^		
Sweet fizzy beverages	Male	75/293 (26%)	129/471 (27%)	0.30	.586
	Female	52/199 (26%)	90/648 (14%)	16.35	.001^a^
	*χ* ^2^	0.02	31.58		
	*p*	.894	.001^a^		
Sugar‐free fizzy beverages	Male	55/293 (19%)	75/471 (16%)	1.04	.308
	Female	37/199 (19%)	83/648 (13%)	4.19	.041^a^
	*χ* ^2^	0.00	2.18		
	*p*	.960	.140		
Sweetened juices	Male	64/293 (22%)	102/471 (22%)	0.00	.951
	Female	51/199 (26%)	105/648 (16%)	9.00	0.003^a^
	*χ* ^2^	0.95	5.38		
	*p*	.330	.020^a^		
Crisps	Male	35/293 (12%)	51/471 (11%)	0.23	0.635
	Female	25/199 (13%)	30/648 (5%)	15.78	0.001^a^
	*χ* ^2^	0.04	15.61		
	*p*	.837	.001^a^		

a Statistical significance *p* < .05.

The final analysis focused on the relationship between excessive computer use and oral health. Excessive computer use decreased the likelihood of a healthy periodontium (71% vs. 64%, *p* = .011) and increased the risk of gingival bleeding (35% vs. 29%, *p* = .009). Even after adjusting for SES (AOR model), excessive computer users were less likely to have a healthy periodontium. The probability of experiencing oral pain resulting in an absence from school was also higher among adolescents who spent too much time in front of the computer (10% vs. 6%, *p* = .013; Table 4).

**Table 4:** Association between excessive computer use with parameters for the condition of the dentition

Parameters	>3 h/d	≤3 h/d	OR	p	CI^a^	AOR	p	CI^a^
DMFT > 0	457/492	1044/1119	0.94	.763	0.62 1.42	1.03	.877	0.68
	93%	93%						1.58
Healthy periodontium	317/492	792/1119	0.75	.011^b^	0.60	0.79	.042^b^	0.63
	64%	71%			0.94			0.99
Bleeding after probing the pocket	174/492	323/1119	1.35	.009^b^	1.08	1.28	.037^b^	1.01
	35%	29%			1.69			1.60
Gingival pockets ≥4 mm	8/492	14/1119	1.31	.550	0.54	1.30	.566	0.53
	2%	1%			3.13			3.19
Oral pain/discomfort	121/492	249/1119	1.14	.304	0.89	1.35	.024^b^	1.04
	25%	22%			1.46			1.74
Absence at school due to oral pain	49/492	72/1119	1.61	.013^b^	1.10	1.80	.003^b^	1.22
	10%	6%			2.35			2.65

Abbreviations: AOR, adjusted odds ratio for socioeconomic factors; CI, confidence interval; DMFT, decayed, missing, and filled teeth; OR, odds ratio.

a CI: lower and upper bound, 95%.

b Statistical significance *p* < .05.

A healthy periodontium was more likely to be seen among both males and females who spent less time in front of a computer screen. However, these differences were not statistically significant (males *p* = .098, females *p* = .236). Although the symptoms of oral pain and an absence at school due to oral pain in both groups were more frequently observed among females than males (Table 5).

**Table 5:** Association between excessive computer use with parameters for the condition of the dentition according to gender

Computer usage every day					
Parameters	Sex	>3 h/d	≤3 h/d	*χ* ^2^	*p*
Healthy periodontium	Male	180/293 (61%)	317/471 (67%)	2.74	.098
	Female	137/199 (69%)	474/648 (73%)	1.40	.236
	χ^2^	2.84	4.50		
	p	0.092	0.034^a^		
Bleeding after probing the pocket	Male	113/293 (39%)	153/471 (32%)	2.94	.086
	Female	61/199 (31%)	170/648 (26%)	1.50	.221
	χ^2^	3.25	5.19		
	p	0.072	0.023^a^		
Gingival pocket ≥4 mm	Male	4/293 (1%)	5/471 (1%)	0.14	.705
	Female	4/199 (2%)	9/648 (1%)	0.39	.533
	χ^2^	0.31	0.24		
	p	0.579	0.627		
Oral pain/discomfort	Male	51/293 (17%)	64/471 (14%)	2.06	.151
	Female	70/199 (35%)	185/648 (29%)	3.18	.075
	χ^2^	20.18	35.29		
	p	0.001^a^	0.001^a^		
Absence at school due to oral pain	Male	21/293 (7%)	21/471 (4%)	2.55	0.110
	Female	28/199 (14%)	51/648 (8%)	6.92	0.009^a^
	χ^2^	6.30	5.27		
	p	0.012^a^	0.022^a^		

a Statistical significance *p* < .05.

Adolescents who used the computer excessively had significantly more unfilled cavities when compared with 18‐year‐olds without these behaviours (2.2 vs. 1.9, *p* = .047). The above analysis was also carried out with attention to gender. It was found that decayed teeth were slightly more common among both males and females who use the computer too much, but these differences were not statistically significant (Table 6).

**Table 6:** Relationship between computer usage every day and DMFT/DMFS index

	Total		Males		Females		Comparisons males vs. females	
Parameters	> h/d	≤3 h/d	>3 h/d	≤3 h/d	>3 h/d	≤3 h/d	>3 h/d	≤3 h/d
DMFT > 0	457/492 (93%)	1044/1119 (93%)	267/293 (91%)	430/471 (91%)	190/199 (95%)	614/648 (95%)	*χ* ^2^ = 3.40	*χ* ^2^ = 5.22
							*p* = .065	*p* = .022^*^
*χ* ^2^	‐		0.01		0.17			
*p*	‐		.936		.684			
DMFT^#^	6.54 ± 4.42	6.48 ± 4.13	6.33 ± 4.37	6.46 ± 4.31	6.84 ± 4.49	6.50 ± 4.00	*p* = .210	*p* = .873
	*p* = .802		*p* = .700		*p* = .305			
Decayed teeth^#^	2.27 ± 3.07	1.97 ± 2.69	2.61 ± 3.29	2.28 ± 2.90	1.78 ± 2.65	1.75 ± 2.50	*p* = .003^*^	*p* = .001^*^
	*p* = .047^*^		*p* = .151		*p* = .863			
Missing teeth^#^	0.13 ± 0.47	0.13 ± 0.51	0.11 ± 0.45	0.13 ± 0.56	0.16 ± 0.51	0.14 ± 0.47	*p* = .253	*p* = .746
	*p* = .917		*p* = .664		*p* = .575			
Filled teeth^#^	4.13 ± 3.63	4.38 ± 3.42	3.61 ± 3.36	4.05 ± 3.36	4.90 ± 3.88	4.61 ± 3.45	*p* < .001^*^	*p* = .007
	*p* = .199		*p* = .083		*p* = .322			
DMFS^#^	9.67 ± 8.57	9.77 ± 8.07	9.35 ± 8.24	9.84 ± 8.45	10.13 ± 9.03	9.72 ± 7.80	*p* = .322	*p* = .806
	*p* = .818		*p* = .437		*p* = .536			

Abbreviations: DMFS, decayed, missing, and filled surfaces; DMFT, decayed, missing, and filled teeth.

*x* ± *SD*.

Statistical significance *p* < .05.

## 4 DISCUSSION

Usually in research, attention has been paid to the common risky behaviours of adolescents including cigarette smoking, drinking alcohol, inappropriate diet, and their relationship to oral health. (Ciecierski, Cherukupalli, & Weresa, 2011; Panatto et al., 2013; Rodrigues, Padez, Ferreira, Gonçalves‐Silva, & Pereira, 2016) However, the excessive use of computers has recently become another serious problem. Studies conducted in a number of countries have revealed that dysfunctional internet behaviour affects around 40% of adolescents aged 14–18.(Lićwinko et al., 2011; Mark et al., 2006; Rodrigues et al., 2016) There is much evidence that excessive computer use generates both physical and psychological problems. (Kim et al., 2010; Marshall et al., 2004; Peltzer et al., 2014) Nevertheless, the relationship between the excessive use of computers and oral health has not been studied in the youth of Poland. Since the political changes of 1989, Poland has experienced economic‐social change, which have significantly influenced health behaviour among young people. Therefore, the current study sought to include 18‐year‐olds from the whole the country, living in both rural and urban areas. As seen in the economic data for 2014, although these areas have different specificities, the economic status of the population of these areas does not differ significantly from each other. (Łącka, 2017) This indicates that the selection of the areas of the country, which took part in the study, did not affect the results or their analysis. It is also worth noting that the number of schools participating in the research was satisfactory and allowed the investigators to draw general conclusions about 18‐year‐olds in Poland. The willingness to participate in the study was a good sign that both school staff and the 18‐year‐olds understood and accepted the need for research into the impact of computer use on the health of the oral cavity.

The present study showed that excessive computer use was more frequent in males than females. This finding seems to be in agreement with other studies. Among the adolescents from Canada, the prevalence of girls who watched more than 2 h/d or more was lower (59%) than in boys (66%).(Mark et al., 2006) Similar results came from the Korean population where 69% of boys and 30% of girls excessively used the computer.(Park & Lee, 2018) Also, long‐time computer use (>3 h/d) has been reported to be found more often in men than women in the Polish group.(Lićwinko et al., 2011) The gender difference in computer use may be influenced by many factors. One of the most frequently mentioned factors is related to parental expectations towards their children. Parents have different hopes for their sons' and their daughters' occupational careers. Men's professions are more often identified with technology and IT than women's professions, which are frequently teachers or nurses. This may make boys spend more time in front of a computer than girls.(Shashaani, 1997)

It was found that excessive computer use was associated with less frequent tooth brushing and use of dental floss. The AOR model confirmed that regular tooth brushing does not depend on the SES, whereas the use of dental floss was found to be to be largely dependent on the SES. This observation has been confirmed by other studies. It is known that the likelihood of daily flossing is higher among persons with a higher level of education (Ronis, Lang, Farghaly, & Passow, 1993) and income,(Farsi, Farghaly, & Farsi, 2004) whereas toothpaste seems to be the main material used for cleaning teeth among families with low SES.

In the present study, there was no significant association between computer use and attendance for dental appointments. Similar results have been observed among the Korean adolescents.(Park & Lee, 2018) Although these authors considered that it might be due to the lack of division into treatment‐related visits and those related only to the routine dental check‐up.(Park & Lee, 2018) Therefore, in the present study, dental appointments were divided into two categories: those for a routine dental check‐up at least once a year and those due a dental emergency (toothache). But in both cases, no statistical correlation was found. This may indicate that excessive computer use does not affect attendance for dental appointments. It seems that young have similar dental attendance patterns to their parents and/or schools (where it is also possible to take advantage of dental care) but it has no socioeconomic background.

The present study confirmed that excessive computer use is associated with an unhealthy diet. In fact, adolescents who spent too much time in front of the computer were more likely to miss breakfast and consume diets high in refined grains, added sugar, and fats. The consumption of these foods has been associated with a higher risk of disease and higher mortality rates.(World Health Organization, 2003) A similar correlation between an increased time spent in front of the TV/computer and unhealthy dietary habits of adolescents has been reported in numerous studies.(Petersen, Jiang, Peng, Tai, & Bian, 2008; Rey‐López et al., 2011; Vereecken, Todd, Roberts, Mulvihill, & Maes, 2005) But in the present study, this correlation was not influenced by a confounding factor, which was SES. On the other hand, the analysis of the consumption of fruit and vegetables was different, in that the time spent in front of a computer did not affect the amount of fruit and vegetables consumed but was affected by SES. Diets high in fresh vegetables and fruits tended to be more frequent among people with a higher SES. Also, other studies have indicated that higher SES groups are more likely to consume fresh vegetables and fruits not only in higher quantities but in a greater variety.(Ball et al., 2005; Darmon & Drewnowski, 2008)

Excessive use of the computer has been correlated with the gender of youths. Preventive dental behaviours (tooth brushing and flossing) were more frequent among females than males. This result was similar to those from other studies because, generally, tooth brushing or flossing is observed more in females.(Farsi et al., 2004; Ronis et al., 1993) Similar differences were observed in relation to the dietary habits. The differences between females and males who spend less time (≤3 h/d) in front of the screen are particularly clear. Generally, in this case, males have a tendency to have more unhealthy nutritional habits (e.g., more frequently miss breakfast, more frequently consume high energy dense products such as sweet fizzy beverages and sweetened juices). While, these differences between the sexes “disappear” in the case of youths often using the computer (> 3 h/d). In these cases, the nutritional behaviour of males and females was almost identical. This result was influenced by a marked increase in nutritionally negative habits among girls. Similar observations were noted in a previous study,(Rey‐López et al., 2011) which evaluated eating and drinking by adolescents while watching TV.

Some researchers have emphasised that the negative effects of excessive computer use by adolescents is poorer oral health (chipped or broken tooth, toothache, bleeding gums, and bad breath).(Kyung‐Yi & Kang‐Soon, 2018; Park & Lee, 2018) However, these studies did not present DMFT/DMFS indices. In the current study, significantly higher DMFT or DMFS values were not observed in those spending more time on the computer than in individuals without such behaviour. However, decayed teeth values were markedly higher (2.27 ± 3.07 vs. 1.97 ± 2.69), indicating considerable neglect of dental care. This can be explained by the fact that the time spent in front of a computer increases the probability of unhealthy dietary habits and/or oral hygiene.

The current study suggested that excessive time spent on the computer might promote the development of gingivitis. The youths who spent more time in front of the computer showed more bleeding after probing than youths who did not use the computer often. This fact is not the result of a different SES because the statistical significance was observed both before and after using the AOR model. It seems that this phenomenon can be explained by poorer oral hygiene, inappropriate dietary habits, and/or a lack of sleep. Some studies have suggested that excessive use of the Internet can cause a lack of sleep or sleep deprivation.(Besedovsky, Lange, & Born, 2012; Grover, Malhotra, & Kaur, 2015) If sleep deprivation continues the immune system will eventually become vulnerable to infection and together with poor oral hygiene can lead to gingivitis.

## 5 CONCLUSION

The main aim of the research was to test the hypothesis that excessive use of the computer influences inappropriate health habits and changes in the health condition of the teeth and periodontal tissues. This hypothesis was confirmed in this study. Adolescents who used the computer excessively had a significantly higher risk of oral hygiene neglect, unfilled cavities, or an inappropriate diet compared with 18‐year‐olds without these behaviour. However, it is worth noting that not all nutritional behaviour or oral hygiene habits are directly related to excessive computer use. Using dental floss or the consumption of fresh fruits and vegetables are more dependent on socioeconomic status than overuse of the computer. These aspects should be considered when designing educational programs for adolescents promoting a healthy lifestyle and patient‐tailored education in dental surgeries.

## CONFLICT OF INTEREST

The authors have no conflict of interests for publication of this manuscript. All authors have made substantive contribution to this study and/or manuscript, and all have reviewed the final paper prior to its submission.

## AUTHOR CONTRIBUTIONS

D.O‐K. and U.K. contributed to the conception of the manuscript. D.O‐K. and J.T. collected the data. D.G. performed the statistical analysis. D.O‐K., J.T., and U.K. evaluated critically and wrote up of the manuscript. D.O‐K., J.T., U.K., and D.G. subsequently revised the drafts. All authors read and approved the final manuscript.

## Supporting information

Data S1. Annex 1: QUESTIONNAIRE FOR INTERVIEW

Data S2. Annex 2: Record card (by tooth surface)
