# Supplementary material for: Excessive computer use as an oral health risk behaviour in 18‐year‐old youths from Poland: A cross‐sectional study
Source: Clin Exp Dent Res. 2019 May 1;5(3):284–93. doi: 10.1002/cre2.183 (PMC6585579; doi:10.1002/cre2.183)
Supplement: Supplementary file 1 — Data S1. Annex 1: QUESTIONNAIRE FOR INTERVIEW [file CRE2-5-284-s001.doc]

**Annex 1: QUESTIONNAIRE FOR INTERVIEW**

ID number ⬜⬜⬜⬜ city ⬜ village ⬜ Man ⬜ Female ⬜

Your class has been randomly selected for research into oral health. Collective results from this anonymous study will contribute to better dental care for children and adolescents. We ask you to give honest answers. If you do not know how to answer a question, raise your hand and the person leading will come and help you. For each question there is a short instruction (in brackets) indicating the answer method.

| **1.How would you describe the status of your teeth and gums?**  *Insert two "X" characters in the corresponding boxes for teeth and gums.* | **Gums** | **Teeth** |
| --- | --- | --- |
| Very good | ⬜_1.1_ | ⬜_2.1_ |
| Good | ⬜_1.2_ | ⬜_2.2_ |
| Average | ⬜_1.3_ | ⬜_2.3_ |
| Bad | ⬜_1.4_ | ⬜_2.4_ |
| Very bad | ⬜_1.5_ | ⬜_2.5_ |
| I do not know | ⬜_1.6_ | ⬜_2.6_ |

| **2. In the last 12 months, how often have you experienced toothache or any discomfort from the oral cavity?** *Put the "X" sign with the right answer.* | |
| --- | --- |
| Very often | ⬜_1_ |
| Occasionally | ⬜_2_ |
| Rarely | ⬜_3_ |
| Never | ⬜_4_ |
| I do not remember | ⬜_5_ |

| **3. Have you had any problems with your teeth or mouth during the last 12 months?**  *Answer each claim by putting "X" in the right box.* | **Yes** | **No** |
| --- | --- | --- |
| No, I'm satisfied with the appearance of my teeth | ⬜_1.1_ | ⬜_2.1_ |
| Because of the ugly teeth I often avoid smiling | ⬜_1.2_ | ⬜_2.2_ |
| Others laugh at my teeth | ⬜_1.3_ | ⬜_2.3_ |
| Because of toothache or other ailments, I left the day at school | ⬜_1.4_ | ⬜_2.4_ |
| I have trouble eating hard foods | ⬜_1.5_ | ⬜_2.5_ |
| I have trouble chewing | ⬜_1.6_ | ⬜_2.6_ |

| **4. How would you rate your knowledge and skills in the area of caring for your teeth and gums?** | |
| --- | --- |
| Very good/Good | ⬜_1_ |
| Limited (general knowledge) | ⬜_2_ |
| Insufficient/lack | ⬜_3_ |

| **5. Indicate which of the following sentences is true or false, and if you are not sure what to answer - get the "X" in the third column.** *Respond to each statement*. | **True** | **False** | **I do not know** |
| --- | --- | --- | --- |
| In children and adolescents, tooth decay develops much faster than in adults. | ⬜_1.1_ | ⬜_2.1_ | ⬜_3.1_ |
| If parents have a lot of cavities, their children will also have a lot of cavities, regardless of whether they care for them or not. | ⬜_1.2_ | ⬜_2.2_ | ⬜_3.2_ |
| Fluoride compounds penetrate the tooth enamel making them resistant to decay. | ⬜_1.3_ | ⬜_2.3_ | ⬜_3.3_ |
| Eaten after an apple supper can replace the evening cleaning of teeth. | ⬜_1.4_ | ⬜_2.4_ | ⬜_3.4_ |
| There are dental surfaces and spaces that can not be cleaned with a brush, therefore the dental floss should be additionally used. | ⬜_1.5_ | ⬜_2.5_ | ⬜_3.5_ |
| Frequent snacking between meals (breakfast first and second, dinner, afternoon tea and dinner) promotes the development of dental caries. | ⬜_1.5_ | ⬜_2.6_ | ⬜_3.6_ |
| Caring for milk teeth is not important, because the milk teeth are replaced by permanent teeth. | ⬜_1.7_ | ⬜_2.7_ | ⬜_3.7_ |
| In addition to daily used pastes containing small amounts of fluorine, special preparations with significantly higher concentrations of fluorine compounds are available. | ⬜_1.8_ | ⬜_2.8_ | ⬜_3.8_ |
| Fruit juices and sodas can damage your teeth. | ⬜_1.9_ | ⬜_2.9_ | ⬜_3.9_ |
| Children and adolescents should report to the dentist at least once a year. | ⬜_1.10_ | ⬜_2.10_ | ⬜_3.10_ |

| **6. From what source did you learn the most, how to care for your teeth and gums?** *Indicate one answer.* | |
| --- | --- |
| Dentist or dental assistant | ⬜_1_ |
| Teachers at school | ⬜_2_ |
| Internet, television, radio | ⬜_3_ |
| Parents, siblings, grandparents | ⬜_4_ |
| Youth magazines, posters, leaflets | ⬜_5_ |
| Classmate, friends | ⬜_6_ |
| Other source - give what | ⬜_7_ |
| None of the above-mentioned sources have received adequate dental knowledge | ⬜_8_ |

| **7. How often do you clean your teeth?** | |
| --- | --- |
| Never | ⬜_1_ |
| Once a month | ⬜_2_ |
| 2-3 times a month. | ⬜_3_ |
| Weekly | ⬜_4_ |
| 2-6 times a week | ⬜_5_ |
| Once a day | ⬜_6_ |
| 2 or more times a day | ⬜_7_ |

| **8. Do you use any of the following to clean your tooth?** *Respond to each statement*.*.* | **Yes** | **No** |
| --- | --- | --- |
| Toothbrush for teeth | ⬜_1.1_ | ⬜_2.1_ |
| Wooden toothpick | ⬜_1.2_ | ⬜_2.2_ |
| Plastic toothpick | ⬜_1.3_ | ⬜_2.3_ |
| Interdental brush | ⬜_1.4_ | ⬜_2.4_ |
| Dental floss | ⬜_1.5_ | ⬜_2.5_ |
| Other | ⬜_1.6_ | ⬜_2.6_ |
| If you have ticked "Yes", next to "Other", please specify what?.............................................................. | | |
| Do you use toothpaste to clean your teeth? | ⬜_1.7_ | ⬜_2.7_ |
| If you use a toothpaste, does it contain fluoride? | ⬜_1.8_ | ⬜_2.8_ |
| Do you use mouthwash? | ⬜_1.9_ | ⬜^2.9^ |
| Do you use sugar substitutes? (e.g. xylitol, stevia) | ⬜_2.0_ | ⬜_3.0_ |

**9. Enter how many hours a day do you spend on a computer on average?**....................................................

| **10. How often do you eat or drink any of the following food / drinks?** *Address the products in each row*. | Several times a day | Every day | A few times a week | Weekly | Several times a month | Rarely/ Never |
| --- | --- | --- | --- | --- | --- | --- |
| Fresh fruit and vegetables | ⬜_1.1_ | ⬜_2.1_ | ⬜_3.1_ | ⬜_4.1_ | ⬜_5.1_ | ⬜_6.1_ |
| Biscuits, cookies, cakes | ⬜_1.2_ | ⬜_2.2_ | ⬜_3.2_ | ⬜_4.2_ | ⬜_5.2_ | ⬜_6.2_ |
| Donuts, cakes, glazed bread rolls | ⬜_1.3_ | ⬜_2.3_ | ⬜_3.3_ | ⬜_4.3_ | ⬜_5.3_ | ⬜_6.3_ |
| Jam / honey | ⬜_1.4_ | ⬜_2.4_ | ⬜_3.4_ | ⬜_4.4_ | ⬜_5.4_ | ⬜_6.4_ |
| Chewing gum containing sugar | ⬜_1.5_ | ⬜_2.5_ | ⬜_3.5_ | ⬜_4.5_ | ⬜_5.5_ | ⬜_6.5_ |
| Chewing gum without sugar | ⬜_1.5_ | ⬜_2.5_ | ⬜_3.5_ | ⬜_4.5_ | ⬜_5.5_ | ⬜_6.5_ |
| Sweets / candy | ⬜_1.6_ | ⬜_2.6_ | ⬜_3.6_ | ⬜_4.6_ | ⬜_5.6_ | ⬜_6.6_ |
| Sodium-sweetened beverages (e.g. Cola Cola, lemonade) | ⬜_1.7_ | ⬜_2.7_ | ⬜_3.7_ | ⬜_4.7_ | ⬜_5.7_ | ⬜_6.7_ |
| Soft soda drinks | ⬜_1.7_ | ⬜_2.7_ | ⬜_3.7_ | ⬜_4.7_ | ⬜_5.7_ | ⬜_6.7_ |
| Energy drinks | ⬜_1.7_ | ⬜_2.7_ | ⬜_3.7_ | ⬜_4.7_ | ⬜_5.7_ | ⬜_6.7_ |
| Tea with sugar | ⬜_1.8_ | ⬜_2.8_ | ⬜_3.8_ | ⬜_4.8_ | ⬜_5.8_ | ⬜_6.8_ |
| Coffee with sugar | ⬜_1.9_ | ⬜_2.9_ | ⬜_3.9_ | ⬜_4.9_ | ⬜_5.9_ | ⬜_6.9_ |
| Sweetened juices | ⬜_1.10_ | ⬜_2.10_ | ⬜_3.10_ | ⬜_4.10_ | ⬜_5.10_ | ⬜_6.10_ |
| Crisps | ⬜_1.11_ | ⬜_2.11_ | ⬜_3.11_ | ⬜_4.11_ | ⬜_5.11_ | ⬜_6.11_ |
| Mineral water | ⬜_1.11_ | ⬜_2.11_ | ⬜_3.11_ | ⬜_4.11_ | ⬜_5.11_ | ⬜_6.11_ |
| Cheese, natural yoghurt, milk | ⬜_1.11_ | ⬜_2.11_ | ⬜_3.11_ | ⬜_4.11_ | ⬜_5.11_ | ⬜_6.11_ |

| **11. Please select which meals during the day (daily) you eat?** | |
| --- | --- |
| 1^st^ breakfast | ⬜_1.1_ |
| A snack after breakfast | ⬜_1.2_ |
| 2^nd^ breakfast | ⬜_1.3_ |
| A snack after the second breakfast | ⬜_1.4_ |
| Dinner | ⬜_1.5_ |
| A snack after lunch | ⬜_1.6_ |
| Afternoon tea | ⬜_1.7_ |
| Snack after tea | ⬜_1.8_ |
| Supper | ⬜_1.9_ |
| A snack after dinner | ⬜_1.10_ |
| A meal before bedtime | ⬜_1.11_ |

| **12. How often do you smoke cigarettes?** | | | | | |
| --- | --- | --- | --- | --- | --- |
| Every day | A few times a week | Weekly | Several times a month | Rarely | Never |
| ⬜_1_ | ⬜_2_ | ⬜_3_ | ⬜_4_ | ⬜_5_ | ⬜_6_ |

| **13. How often do you drink alcohol?** | | | | | |
| --- | --- | --- | --- | --- | --- |
| Every day | A few times a week | Weekly | Several times a month | Rarely | Never |
| ⬜_1_ | ⬜_2_ | ⬜_3_ | ⬜_4_ | ⬜_5_ | ⬜_6_ |

| **14. How many times have you been at the dentist during the last 12 months?** *Tick "X" in only one window.*  *If you have not been to the dentist in the last 12 months or two years, quit question 14 and go to question 15.* | |
| --- | --- |
| 1 time | ⬜_1_ |
| 2 times | ⬜_2_ |
| 3 times | ⬜_3_ |
| 4 times or more | ⬜_4_ |
| I have not been to the dentist during the last 12 months | ⬜_5_ |
| I have not been to the dentist for two years | ⬜_6_ |
| I do not remember when I was at the dentist | ⬜_7_ |

| **15. During the last visit or previous visits, have the dentist or dental assistant.** *Answer each claim by putting an "X" in the appropriate box.* | **Yes** | **No** | **I do not remember** |
| --- | --- | --- | --- |
| She/He explained exactly what is the state of your teeth and gums. | ⬜_1.1_ | ⬜_2.1_ | ⬜_3.1_ |
| She/He specified how often you should report for a control visit. | ⬜_1.2_ | ⬜_2.2_ | ⬜_3.2_ |
| She/He instructed or reminded you how to properly clean your teeth. | ⬜_1.3_ | ⬜_2.3_ | ⬜_3.3_ |
| She/He instructed how to use dental floss. | ⬜_1.4_ | ⬜_2.4_ | ⬜_3.4_ |
| She/He indicated what toothbrush you should clean your teeth. | ⬜_1.5_ | ⬜_2.5_ | ⬜_3.5_ |
| She/He indicated what paste you should use. | ⬜_1.6_ | ⬜_2.6_ | ⬜_3.6_ |
| She/He recommended specific preparations containing fluorine. | ⬜_1.7_ | ⬜_2.7_ | ⬜_3.7_ |
| She/He gave instructions on a diet limiting caries. | ⬜_1.8_ | ⬜_2.8_ | ⬜_3.8_ |

| **16. What was the reason for your last visit to the dentist?** | |
| --- | --- |
| Pain or discomfort associated with teeth, gums or mouth. | ⬜ _1.1_ |
| Treatment / continuation of treatment. | ⬜ _1.2_ |
| Review / check-up. | ⬜ _1.3_ |
| Tooth cleaning. | ⬜_1.4_ |
| I do not remember. | ⬜ _1.5_ |

| **17. Have your parents/guardians or you paid for the last visit to the dentist?** |  |
| --- | --- |
| Yes, they paid | ⬜ _1.1_ |
| No, they did not pay | ⬜ _1.2_ |
| I do not remember | ⬜ _1.3_ |

| **18. It happens that we notice the necessity of applying to the dentist, but for various reasons postpone the appointment. What are the reasons for postponing your visit?** *Answer each sentence*. | **Yes** | **No** |
| --- | --- | --- |
| I do not postpone the visit, whenever I feel the need, I report to the dentist. | ⬜_1.1_ | ⬜_2.1_ |
| *If you do not postpone the visit, i.e. you have marked 'Yes', leave the rest of the question.* | | |
| I postpone reporting to the dentist because: *Respond to each claim, putting "X" in the appropriate box* | **Yes** | **No** |
| I can not afford treatment from a private dentist, and a dentist working within the National Health Fund does not meet my expectations (she/he sets long deadlines or does not have good materials, or does not do well, or is not polite). *emphasize the right answer*. | ⬜_1.2_ | ⬜_2.2_ |
| I can not afford a private dentist treatment, and a dentist working within the National Health Fund does not perform the procedures my teeth or gums require. | ⬜_1.3_ | ⬜_2.3_ |
| I'm afraid of the pain a dentist can do to me. | ⬜_1.4_ | ⬜_2.4_ |
| I think my teeth problems will subside. | ⬜_1.5_ | ⬜_2.5_ |
| I am postponing the visit, because I suppose that the dentist will not help me much. | ⬜_1.6_ | ⬜_2.6_ |
| I do not care too much about the state of my teeth, I have other more important problems. | ⬜_1.7_ | ⬜_2.7_ |
| Colleagues having problems with their teeth also postpone the application to the dentist. | ⬜_1.8_ | ⬜_2.8_ |
| I have another reason to postpone the visit - give it what:………………………. | | |

| **19. What education your parents or guardians have?** | **Father or guardian** | **Mother or guardian** |
| --- | --- | --- |
| Basic | ⬜_1.1_ | ⬜_2.1_ |
| Professional | ⬜_1.2_ | ⬜_2.2_ |
| Average | ⬜_1.3_ | ⬜_2.3_ |
| Higher or incomplete higher | ⬜_1.4_ | ⬜_2.4_ |
| I do not know | ⬜_1.5_ | ⬜_2.5_ |

| **20. What is the financial situation of your family (compared to other families)?** | |
| --- | --- |
| Below the average | ⬜_1_ |
| Average | ⬜_2_ |
| Above average | ⬜_3_ |
| I do not know | ⬜_4_ |

Thank you for honestly filling out the questionnaire. Check if you have answered all the questions!
***
If you would like to provide something more about dental care, please do it here.

………………………………………………………………………………………………………………………………………………………………………………………………………………………………………………………………………………………………………………………………………………………………………........................................................................................................................................................................................................................
