# Supplementary material for: Excessive computer use as an oral health risk behaviour in 18‐year‐old youths from Poland: A cross‐sectional study
Source: Clin Exp Dent Res. 2019 May 1;5(3):284–93. doi: 10.1002/cre2.183 (PMC6585579; doi:10.1002/cre2.183)
Supplement: Supplementary file 2 — Data S2. Annex 2: Record card (by tooth surface) [file CRE2-5-284-s002.doc]

# Annex 2: Record card (by tooth surface)

Age: 18 years old

|  |  |  |  |  |
| --- | --- | --- | --- | --- |

Identification Number

|  |  |
| --- | --- |

Region Location: Urban ⬜ Rural ⬜ Male **⬜** Female **⬜**

Name, surname:.................................................. date of examination: year.......month.......day........

Name of the examining doctor........................................................................................

**Dental status by tooth surface**

|  | 17 | 16 | 15 | 14 | 13 | 12 | 11 | 21 | 22 | 23 | 24 | 25 | 26 | 27 |
| --- | --- | --- | --- | --- | --- | --- | --- | --- | --- | --- | --- | --- | --- | --- |
| **O** |  |  |  |  | X | X | X | X | X | X |  |  |  |  |
| **M** |  |  |  |  |  |  |  |  |  |  |  |  |  |  |
| B |  |  |  |  |  |  |  |  |  |  |  |  |  |  |
| D |  |  |  |  |  |  |  |  |  |  |  |  |  |  |
| L |  |  |  |  |  |  |  |  |  |  |  |  |  |  |

Status
0 = healthy

1 = caries

2 = filled w/caries

3 = filled no/caries

4 = missing due to caries

5 = missing for another reason

6 = fissure sealant

7 = fixed partial denture/crown, abutment, veneer, implant

# 8 = unerupted

9 = not recorded

|  | 47 | 46 | 45 | 44 | 43 | 42 | 41 | 31 | 32 | 33 | 34 | 35 | 36 | 37 |
| --- | --- | --- | --- | --- | --- | --- | --- | --- | --- | --- | --- | --- | --- | --- |
| O |  |  |  |  | X | X | X | X | X | X |  |  |  |  |
| M |  |  |  |  |  |  |  |  |  |  |  |  |  |  |
| B |  |  |  |  |  |  |  |  |  |  |  |  |  |  |
| D |  |  |  |  |  |  |  |  |  |  |  |  |  |  |
| L |  |  |  |  |  |  |  |  |  |  |  |  |  |  |

Status

0 = lack of healing needs

1= filling on the 1 surface

2 = filling on the 2 surfaces

3 = filling on more than 2 surfaces.

4 = prevention

5 = pulp treatment

6 = extraction

7 = other

**Health needs**

| 17 | 16 | 15 | 14 | 13 | 12 | 11 | 21 | 22 | 23 | 24 | 25 | 26 | 27 |
| --- | --- | --- | --- | --- | --- | --- | --- | --- | --- | --- | --- | --- | --- |
|  |  |  |  |  |  |  |  |  |  |  |  |  |  |
|  |  |  |  |  |  |  |  |  |  |  |  |  |  |
| 47 | 46 | 45 | 44 | 43 | 42 | 41 | 31 | 32 | 33 | 34 | 35 | 36 | 37 |

0 = no treatment needed

1 = preventive or routine treatment needed

2 = prompt treatment (includiong scaling) needed

3 = immediate treatment needed due to pain or infection of dental and/or oral origin

4 = referred for comprehensive evaluation or medical treatment (systemic onditions)

No of teeth

**Intervention urgency**

**Dental trauma**

No. of teeth

0 = no sign of injury

1 = treated injury

2 = enamel fracture only

3 = enamel + dentine fracture

4 = pulp involvement

5 = missing tooth due to trauma

6 = other damage

9 = excluded tooth

No. of teeth

0 = no sign of erosion

1 = enamel lesion

2 = dentinal lesion

3 = pulp involvement

Sextants in the maxilla:………………………………………………

Sextants in the mandible:……………………………………………..

**Dental erosion**

**Periodontal status**

Bleeding

0 = absence of condition

1 = presence of condition

Pocket

0 = absence of condition

1 = pocket 4-5mm

2 = pocket 6mm or more

9 = excluded tooth

X = tooth not present

Calculus

0 = absence of condition

1 = presence of condition

|  | 17 | 16 | 15 | 14 | 13 | 12 | 11 | 21 | 22 | 23 | 24 | 25 | 26 | 27 |
| --- | --- | --- | --- | --- | --- | --- | --- | --- | --- | --- | --- | --- | --- | --- |
| bleeding |  |  |  |  |  |  |  |  |  |  |  |  |  |  |
| pocket |  |  |  |  |  |  |  |  |  |  |  |  |  |  |
| calculus |  |  |  |  |  |  |  |  |  |  |  |  |  |  |

|  | 47 | 46 | 45 | 44 | 43 | 42 | 41 | 31 | 32 | 33 | 34 | 35 | 36 | 37 |
| --- | --- | --- | --- | --- | --- | --- | --- | --- | --- | --- | --- | --- | --- | --- |
| bleeding |  |  |  |  |  |  |  |  |  |  |  |  |  |  |
| pocket |  |  |  |  |  |  |  |  |  |  |  |  |  |  |
| calculus |  |  |  |  |  |  |  |  |  |  |  |  |  |  |

**Loss of attachment**

0 = 0-3mm

1 = 4-5 mm CEJ with black band

2 = 6-8 mm CEJ between upper limit of blank band and 8.5mm ring

3 = 9-11 mm CEJ between 8,5-11.5mm ring

4 ≥ 12 mm CEJ beyond 11.5mm ring

X = excluded tooth

9 = not recorded

**Oral mucosal lesions**

|  | Presence | Absence | Location |
| --- | --- | --- | --- |
| No abnormal condition |  |  |  |
| Malignant tumor (oral cancer) |  |  |  |
| Leukoplakia |  |  |  |
| Lichen planus |  |  |  |
| Ulceration (aphthous herpetic, traumatic) |  |  |  |
| Acute necrotizing ulcerative gingivitis |  |  |  |
| Candidiasis |  |  |  |
| Abscess |  |  |  |
| Other condition |  |  |  |
| Not recorded |  |  |  |

| 17/16 | 11 | 26/27 |
| --- | --- | --- |
|  |  |  |
|  |  |  |
| 46/47 | 31 | 36/37 |

**Denture(s)**

| upper | lower |
| --- | --- |
|  |  |
|  |  |

0 = no dentures

1 = partial dentures

Status

0 = normal

1 = opacity spilled

2 = opacity limited

3= pit-type defects

4 = furrow-type defects

5 = hypoplasia on the surface

6 = hypoplasia on the incisal

7 = discoloured enamel (not associated with opacity)

2 = complete dentures

9 = not recorded

**Enamel developmental defects**

| 17 | 16 | 15 | 14 | 13 | 12 | 11 | 21 | 22 | 23 | 24 | 25 | 26 | 27 |
| --- | --- | --- | --- | --- | --- | --- | --- | --- | --- | --- | --- | --- | --- |
|  |  |  |  |  |  |  |  |  |  |  |  |  |  |
|  |  |  |  |  |  |  |  |  |  |  |  |  |  |
| 47 | 46 | 45 | 44 | 43 | 42 | 41 | 31 | 32 | 33 | 34 | 35 | 36 | 37 |

**Presence of MIH (Molar incisor hypomineralization)**

0 = presence

1 = absence

No. of teeth

**Enamel fluorosis**

Severity

0 = normal No. of teeth

1 = questionable

2 = very mild

3 = mild

4 = moderate

5 = severe

8 = excluded

9 = not recorded
